# Supplementary material for: Do Social Ties Moderate the Association between Childhood Maltreatment and Gratitude in Older Adults? Results from the NEIGE Study
Source: Int J Environ Res Public Health. 2021 Oct 21;18(21):11082. doi: 10.3390/ijerph182111082 (PMC8582950; doi:10.3390/ijerph182111082)
Supplement: Supplementary file 1 [file ijerph-18-11082-s001.zip › ijerph-1394269-supplementary.pdf]

International Journal of
Environmental Research and Public Health

MDPI

Supplementary Table S1. Correlation matrix among variables (n=524).

		1	2	3	4	5	6	7	8	9	10	11	12	13
1	Gratitude scale	1												
2	Physical abuse	0.02	1											
3	Emotional neglect	**-0.10**	0.03	1										
4	Emotional abuse	<0.01	**0.38**	0.05	1									
5	Neighborhood tie	**0.15**	-0.01	-0.03	-0.06	1								
6	Frequency of meeting friends	**-0.15**	<0.01	0.02	0.01	**-0.18**	1							
7	Sex	0.07	-0.07	-0.05	-0.08	0.05	**-0.20**	1						
8	Age	0.00	-0.04	0.00	-0.02	0.05	0.02	0.00	1					
9	Economic hardship	-0.03	0.03	0.05	**0.12**	0.07	0.03	**-0.14**	0.03	1				
10	Education	0.03	0.05	-0.04	0.06	**-0.17**	<0.01	**-0.22**	**-0.28**	**-0.13**	1			
11	Current depressive symptoms (GDS score)	**-0.21**	-0.01	0.02	0.06	**-0.18**	**0.18**	0.07	0.06	**0.10**	0.01	1		
12	Annual income	0.03	-0.01	-0.02	0.02	0.00	-0.02	-0.06	**-0.16**	-0.09	**0.21**	**-0.11**	1	
13	Longest occupation	-0.02	0.04	-0.03	**0.15**	-0.08	-0.02	**-0.19**	-0.08	-0.06	**0.28**	0.02	0.06	1
14	Marital status	0.04	0.03	-0.03	-0.04	**0.09**	-0.07	**0.20**	0.07	0.02	-0.06	0.06	-0.08	-0.08

GDS = geriatric Depression Scale.
Bold signifies p<0.05.

Supplementary Table S2. Gratitude scale scores of participants (n=524).

**Items**	**All**		**Sex**				
			**Male (n=246)**		**Female (n=278)**		**p-value[a]**
	**mean**	**SD**	**mean**	**SD**	**mean**	**SD**	
I have so much in life to be thankful for (1-7)	6.22	1.04	6.15	1.05	6.27	1.03	0.19
I am grateful to a wide variety of people (1-7)	6.36	0.94	6.28	0.93	6.43	0.95	0.06
Gratitude scale (1-7)	6.29	0.93	6.22	0.95	6.35	0.91	0.09

[a] Sex differences were tested using t-test.

Supplementary Table S3. Associations of childhood emotional neglect with gratitude according to the levels of neighborhood tie and frequency of meeting friends among Japanese older adults (n=524).

	n	**Coefficient (95%CI)**
Neighborhood tie		
High	226	0.22 (-0.13 to 0.57)
Low	298	**-0.57 (-0.91 to -0.23)**
Frequency of meeting friends		
High	312	-0.08 (-0.38 to 0.23)
Middle	125	-0.28 (-0.80 to 0.24)
Low	87	**-1.07 (-1.87 to -0.27)**

CI = confidence interval.
Model: Adjusted for age, sex, childhood other environment (economic hardship and education), and current depressive symptoms.
Bold signifies p<0.05.
